# Organic acids target key virulence factors of *Aeromonas hydrophila* to reduce infection *in vitro*

**DOI:** 10.3389/fmicb.2026.1809130

**Published:** 2026-05-12

**Authors:** Florica Morariu, Igori Balta, Lavinia Stef, Ioan Pet, Sorin Morariu, Nicolae Corcionivoschi, Ducu Stef

**Affiliations:** 1 Faculty of Bioengineering of Animal Resources, University of Life Sciences King Mihai I from Timisoara, Timisoara, Romania; 2 Faculty of Veterinary Medicine, University of Life Sciences King Mihai I from Timisoara, Timisoara, Romania; 3 Bacteriology Branch, Veterinary Sciences Division, Agri-Food and Biosciences Institute, Northern Ireland, Belfast, United Kingdom; 4 Academy of Romanian Scientists, Bucharest, Romania; 5 Faculty of Food Engineering, University of Life Sciences King Mihai I from Timisoara, Timisoara, Romania

**Keywords:** *Aeromonas hydrophila*, anti-virulence, organic acids, oxidative stress, virulence factors

## Abstract

This study investigates the effects of the organic acid mixture AuraAqua (Aq) on the pathogenicity of *Aeromonas hydrophila* in primary gut cells of shrimp (SGP) and tilapia (TGP). *A. hydrophila*, an important pathogen in aquaculture, damages gut barrier integrity and promotes systemic infection with a significant economic impact. Organic acids are proposed as antibiotic alternatives, and we aimed to investigate their impact as a mixture AuraAqua (Aq—5% maltodextrin, 1% sodium chloride, 42% citric acid, 18% sodium citrate, 10% silica, 12% malic acid, 9% citrus extract, and 3% olive extract) on the growth, virulence traits, and host cell protection during *A. hydrophila* infection SGP and TGP cells. Aq exhibited an MIC of 1% and an MBC of 3%. Sub-inhibitory concentrations significantly reduced adhesion and invasion in SGP and TGP cells, preserved TEER, and decreased LDH release, indicating reduced cytotoxicity and barrier disruption. Aq impaired motility and biofilm formation in a dose-dependent manner. Transcriptional analysis showed downregulation of *hcp1, act, fla, hlyA* and *acg*, whereas *TonB*, *aerA* and alt were largely unaffected. Aq also strongly reduces capsular polysaccharide production both in vitro and during infection, which likely weakens immune evasion and persistence. In host cells, Aq significantly decreases oxidative stress markers (H_2_O_2_, SOD, CAT) during infection, indicating a beneficial effect on host responses. Overall, Aq acts as an anti-virulence agent, weakening *A. hydrophila’s* ability to colonize and damage gut epithelia while reducing host oxidative stress, supporting its use as a non-antibiotic preventive strategy in aquaculture, pending further *in vivo* studies.

## Introduction

*Aeromonas hydrophila* (*A*. *hydrophila*) is a major pathogen in aquaculture, causing motile *Aeromonas* septicemia (MAS) and significant economic losses due to high fish mortality (Semwal et al., 2023; Tao et al., 2026). MAS is described by related syndromes (e.g., hemorrhagic septicemia, fin and tail rot, epizootic ulcerative syndrome), which can lead to explosive outbreaks in cultured fish, shrimp and other aquatic animals with reported mortality rates that may exceed 95% in severe epizootics (Xu et al., 2023; Urgesa et al., 2024). *A. hydrophila* is also a zoonotic and foodborne pathogen, isolated from aquaculture products (e.g., sashimi, oysters, salmon sushi) and capable of causing bacteremia and severe systemic infections in humans by causing sepsis, soft tissue, skin and gastrointestinal infections (Dong et al., 2025a). At the genus level, *Aeromonas* infections (including *A*. *hydrophila* and *A. salmonicida*) cause “economic burdens of dozens of millions of dollars in aquaculture” through mortality, growth loss and associated treatment costs (Lamy et al., 2022). Hypervirulent *A*. *hydrophila* clones such as ST251 have been highlighted as emerging causes of major losses in fish farming and production systems in the United States and China (Xu et al., 2023; Xu et al., 2025). *A. hydrophila* produces multiple virulence factors, e.g., aerolysins, hemolysins, proteases, lipases, toxins and secretion systems—that damage host tissues and suppress immune responses (Abdul Kari et al., 2022). It can survive as free planktonic cells or within biofilms; the latter confer high resistance to disinfectants and antibiotics (Xia et al., 2025).

Control of *A*. *hydrophila* traditionally relies on antibiotics and chemotherapeutics, which carry substantial economic and regulatory burdens, requiring a One Health approach (Lamy et al., 2022). Selection of multidrug-resistant (MDR) strains (e.g., high resistance rates to commonly used drugs such as ampicillin, sulfonamides, and some fluoroquinolones) complicates the therapy, forcing farmers toward more expensive, novel or less accessible antimicrobials and raising the risk of treatment failure (Zhong et al., 2021; Urgesa et al., 2024). A novel antimicrobial peptide (AMP), Spasin from mud crab (*Scylla paramamosain*), disrupted the cell membrane and showed to release ATP and intracellular ROS accumulation (Zhang et al., 2024). Furthermore, Spasin_141–165_ reduced pathogen load in organs, inflammation (IL-1β, TNF, cxcl8, NOS2a, MMP9) in zebrafish without toxicity to host cells and improved the survivability of *A. hydrophila-*infected fish (Zhang et al., 2024). Similarly, the natural antimicrobial ClavF peptide at 100 μL disrupted the pathogen membrane and led to leakage, increased survival/reduced inflammation, and lowered spleen and intestinal damage in grass carp infected with MDR *A. hydrophila* (Peng et al., 2025). PACAP peptide variants acted via membrane permeabilization with low toxicity to fish cells (Méndez et al., 2024). The Epinecidin-1 peptide extracted from fish showed synergistic effects with organic acids, such as lactic acid, following co-treatment, leading to improved attachment, membrane damage, and electromotive force dissipation in *A*. *hydrophila* cells (Li et al., 2024).

Essential oils from satsuma mandarin (*Citrus unshiu*), rich in limonene (≈70%), disrupted the pathogen’s membrane integrity, leading to leakage of intracellular contents (Zhong et al., 2022). Linalool nanoemulsions demonstrated enhanced solubility/stability for aquatic use, and at the MIC and MBC values (0.3125 and 0.625% *v*/*v*, caused membrane disruption, which was confirmed by electron microscopy (Zhong et al., 2021). Turmeric oil has recently and markedly inhibited QS-regulated virulence factors (*aerA*, *ahyI*, and *ahyR*) and alleviated renal injury *in vivo* at sub-MIC concentrations (Dong et al., 2025b). The thymol chemotype of *Lippia graveolens* was highly effective against oxytetracycline-resistant *A. hydrophila* strains (García-Pérez et al., 2024). Likewise, cinnamaldehyde targeted QS/biofilm formation without directly affecting bacterial growth, whereas S-(-)-limonene/R-(+)-limonene inhibited biofilms and showed additive effects with florfenicol (da Silva et al., 2021; Li et al., 2023). Chitosan nanoparticles (CNPs) exhibited direct bactericidal action, causing loss of bacterial architecture, and were effective both *in vitro* and *in vivo* against MDR *A. hydrophila* subsp. *hydrophila* in tilapia, while silver nanoparticles (AgNPs) depicted similar effects with additional downregulation of virulence genes (*aerA, exoU*, and *trh* genes) when combined with H_2_O_2_ (Saad et al., 2024), with the efficiency extended in *P. aeruginosa* and *Vibrio* spp.

The mechanistic understanding of natural antimicrobials, though still evolving, has grown substantially in the last decade, with strong indications that these interventions work not by a single magic-bullet effect, but by modulating multiple antibacterial and host-defense mechanisms. The resulting mode of action is suitable for a complex pathogen, such as *A. hydrophila*, which deploys numerous virulence factors (toxins, biofilms, immune evasion) to cause disease. The aim of this study was to evaluate whether the organic acid mixture AuraAqua (Aq) can act as a non-antibiotic anti-virulence strategy by reducing the pathogenicity of *Aeromonas hydrophila* while protecting host gut epithelial cells. Specifically, the study investigated the effects of Aq on bacterial growth and virulence traits (adhesion, invasion, motility, biofilm formation, virulence gene expression and capsule production) and on host cell integrity and oxidative stress responses in primary shrimp and tilapia gut cells *in vitro*.

## Materials and methods

### Bacterial growth and organic acids mixture

The *Aeromonas hydrophila* strain (ATCC 7966) was grown in McConkey agar (Oxoid, Hampshire, United Kingdom) at 30°C. The natural antimicrobial mixture, AuraAqua (Aq), contains 5% maltodextrin, 1% sodium chloride, 42% citric acid, 18% sodium citrate, 10% silica, 12% malic acid, 9% citrus extract, and 3% olive extract (w/w) (Environtech, Dublin, Ireland). The raw materials were supplied by Bio-Science Nutrition Ireland. Experiments were carried out in triplicate.

### Minimum inhibitory concentration (MIC) and minimum bactericidal concentration (MBC) determination

The twofold tube dilution method was used to determine the lowest concentration of Aq that inhibits bacterial growth (MIC) and the lowest concentration that causes bacterial death (MBC). Aq was diluted (8% down to 0.0078% v/v) in McConkey broth (Thermo Fisher, United Kingdom) and thoroughly vortexed. Overnight bacterial cultures were harvested by centrifugation, washed with PBS, and diluted to 10^6^ CFU/mL in McConkey broth for inoculation. Non-inoculated plates containing the same growth medium were used as negative controls, and tubes inoculated with individual bacterial cultures in McConkey broth without Aq were used as positive controls. Subsequently, the tubes were incubated at 30°C for 48 h. Tubes without visible growth were considered below the MIC. A 100 μL volume was taken from each tube showing no growth and inoculated onto McConkey agar plates at the highest dilution; no microbial growth was considered the MBC. Each assay was performed in triplicate for each strain. To determine the sub-inhibitory concentrations, the two pathogens were exposed to varying concentrations of the antimicrobial. Concentrations that showed no effect on survival and no growth inhibition (with the same growth kinetics as the control) were used for the subsequent experiments.

### Biofilm assay

Biofilm formation in the presence of 0.1, 0.5, 1, and 2% Aq was performed in 96-well polystyrene microtiter plates (Thermo Fisher Scientific, United Kingdom) inoculated with cultures of *A. hydrophila* strain (ATCC 7966), with or without Aq, at a concentration of 10^6^ CFU/mL in McConkey broth medium. A volume of 0.2 mL was added to each well, followed by incubation for 24 h at 37°C. Biofilm was quantified as the amount of crystal violet-stained biomass. Subsequently, 2 mL of methanol was added to each well to fix the adherent bacteria. After 2 min, the methanol was removed, and the plates were washed twice with sterile PBS and air-dried. The next step was crystal violet (CV) staining, in which the volume of 2.25 mL of 0.1% CV solution was added to all wells. After 10 min of dyeing, CV was removed, and the wells were washed twice with PBS and dried. In the last step, 2 mL of 30% glacial acetic acid was added to each well and incubated for 10 min. The content of each plate was moved carefully, without agitation, into a new plate, and the absorbance was measured using a microplate reader (FluoStar Omega, Premier Scientific, Belfast, United Kingdom) at an absorbance of 550 nm, and 30% glacial acetic acid was used as a blank. All the steps were performed at room temperature. The assay was repeated thrice on 3 three separate occasions.

### Primary cell preparation and infection assay

The shrimp gut primary cells (SGP) (Balta et al., 2022) and Tilapia fish gut primary cells (TGP) (Liliana et al., 2024) from laboratory stocks were used as prepared previously. Briefly, the gut tissue was cut into small pieces and gently washed twice by centrifugation (5 min, 150 × g). Five ml 0.25% trypsin at pH 7.4 at room temperature was added for 30–60 min and stirred on a magnetic stirrer, washed twice and cells were put into a 25 cm^2^ plastic culture flask with DMEM growth medium. Cells were further cultured in DMEM media at 28 °C in 24 well plates (Analab, Lisburn, United Kingdom) supplemented with 0.1% DMSO (Thermo-Fisher, UK) and 20% foetal bovine serum (FBS), 100 μg penicillin, 8% shrimp head extract, 6% salt solution, 20 ng epidermal growth factor (Sigma-Aldrich, Gillingham, United Kingdom) and 10 U/mL human recombinant interleukin 2 (Sigma-Aldrich, Gillingham, United Kingdom). Viability was assessed using the trypan blue exclusion method on both floating and attached cells. The SGP and TGP cells were also grown in DMEM (5.5 × 10^5^/well), in 75 cm^2^ flasks (Sigma-Aldrich, Arklow, Ireland, SIAL0641) at 37°C and 5% CO_2_ until 80–90% confluent in 6-well culture plates at pH 7.2. *A. hydrophila* strain (ATCC 7966) was grown in McConkey agar (Oxoid, Hampshire, United Kingdom) at 30°C for 24 followed by dilution in DMEM media (Thermo Fisher, United Kingdom) to ensure an MOI of 100 for cell infection (OD_600_ of ≈ 0.4). The cells were infected for 24 h in DMEM media containing 0.1, 0.5, 1, and 2% AuraAqua (Aq). Adherence was quantified by washing the infected monolayers with PBS followed by exposure to 0.1% Triton X100 in PBS for 15 min at 37°C. Dilutions (10-fold) of infected or control wells were plated onto McConkey agar (Oxoid, Hampshire, United Kingdom) and incubated at 30°C for 24 h, then for 2 days prior to enumeration. To quantify the number of bacteria that invaded SGP and TGP cells, the infected monolayers were washed with DMEM, incubated in fresh medium (2 ml), and then treated with gentamicin (400 μg/mL) to kill extracellular bacteria. The tissue culture plates were then incubated for an additional 3 h at 30°C and washed with fresh DMEM. Cells were lysed by adding 1 ml of 0.1% Triton X-100 in PBS and incubated for 15 min at 30°C. All assays were performed in triplicate and on three separate days. To test whether Aq directly impacts *A. hydrophila* virulence by targeting the pathogen specifically, we have also performed a virulence assay with bacteria exposed only. To achieve the pathogen was grown at the subinhibitory concentration 0.5% Aq in McConkey broth medium for 24 h 30°C. The resulting grown inoculum was then used in the infection assay as described above.

### Cytotoxic lactate dehydrogenase (LDH) release assay

LDH release was used to assess the impact of Aq on the cytotoxic effects of *A. hydrophila* strain (ATCC 7966), as previously described on infected SGP and TGP cells in the presence of 0.1, 0.5, 1, and 2% Aq at 3 h of post-infection. Cytotoxicity was assessed by measuring LDH release from primary shrimp gut epithelial cells (SGP) and primary tilapia gut epithelial cells (TGP) following infection with *A. hydrophila*. The isolation and culture of SGP and TGP cells are described above. Briefly, confluent monolayers of SGP and TGP cells were infected with *A. hydrophila* (ATCC 7966) in the presence or absence of 0.1, 0.5, 1, or 2% AuraAqua. LDH levels were quantified in cell culture supernatants at 3 h post-infection using a commercial cytotoxicity detection kit (Roche, United Kingdom), according to the manufacturer’s instructions. LDH levels were measured in the supernatants of infected SGP and TGP cells using a cytotoxicity detection kit (Roche, Buckinghamshire, United Kingdom) following the manufacturer’s instructions. Readings were taken at 500 nm using a spectrophotometer (FLUOstar Omega, Premier Scientific, Belfast, United Kingdom). Each experiment was carried out in triplicate.

### Transepithelial resistance of cellular tight junctions (TEER)

TGP and SGP cells were plated onto transwells (10^4^; 6.5 mm diameter; 0.4 μm—pore size; Corning, United Kingdom) and grown until apical junctional complexes developed. Transwells were infected apically with either *A. hydrophila* strain (ATCC 7966) only or in the presence of 0.1, 0.5, 1, and 2% Aq. TEER was measured at 3 h post infection using an EVOM X meter connected to an Endohm chamber (World Precision Instruments). Flux assay data are presented as the mean (SD) of triplicate independent samples, each performed three times. The presented results are representative of at least three independent experiments. The mean (SD) of at least three independent experiments for each cell line was calculated.

### RT-qPCR relative gene expression *A. hydrophila* virulence genes

The expression of bacterial virulence genes *hcp1* and *TonB* (Huang et al., 2025) and *aerA, act, fla, alt, hlyA, acg* (Chen et al., 2021) was analyzed. *A. hydrophila* was grown in the presence of 0.1, 0.5, 1, and 2% Aq for 24 h 30°C. Unexposed bacteria served as the control. For bacterial gene expression, total RNA from *A. hydrophila* was extracted using the RNA-easy isolation reagent. by using the RNeasy^®^Plus Mini Kit (Qiagen, United Kingdom). The RNA was reverse transcribed using Transcriptor First Strand cDNA Synthesis Kit (Roche, United Kingdom) according to the manufacturer’s protocol. The mRNA levels were determined by quantitative RT-PCR using QuantiNovaSYBR^®^ Green PCR Kit (Qiagen, United Kingdom) on a LightCycler^®^ 96 (Roche, United Kingdom). The PCR conditions consisted of incubating for 10 min at 95°C followed by 45 cycles of 95°C for 10 s, 55°C for 30 s, and 72°C for 10 s. A total of 5 μL of SYBR Green master mixture was used in each reaction along with 0.5 μL of 10 μM primer mixture, 3 μL of molecular grade water, and 1 μL of cDNA sample. The primers used (Invitrogen, United Kingdom) are described in Table 1. 16S rRNA was used as an internal reference. The relative expression levels of the target genes were calculated using the 2^–ΔΔCt^ method.

**TABLE 1:** Primer sequences.

Gene	Primer sequence	
*hcp1*	F	AAGACGAGATGCTGGTGCAA
	R	TCAGGGTCACTTTCGGCAG
*TonB*	F	ACTGGATCTGACCCTCTCCC
	R	TTTTCTCCGTAGACCACCGC
*aerA*	F	TCCAGCGCATTCA
	R	TCCAGCCTTCGGCAAACG
*act*	F	ATCGTCAGCGACAGCTTCTT
	R	CTCATCCCTTGGCTTGTTGT
*fla*	F	TCCAACCGTYTGACCTC
	R	GMYTGGTTGCGRATGGT
*alt*	F	TGACCCAGTCCTGGCACGGC
	R	GGTGATCGATCACCACCAGC
*hlyA*	F	GGCCGGTGGCCCGAAGATACGGG
	R	GGCGGCGCCGGACGAGACGGGG
*acg*	F	AACAAGCACCCGTTAAGCCAC
	R	ACGTAGTCGAGCCCCTTGAGG

### Capsule polysaccharide (CPS) quantification from *A. hydrophila*

Quantification of CPS levels from *A. hydrophila*, grown in the presence of 0.1, 0.5, 1, and 2% A (24 h), was analyzed following alkaline extraction. *A. hydrophila* was grown in 20 ml McConkey media at 30 °C for 24 h. Bacterial cells were harvested by centrifugation for 15 min at 3220 × g at 4°C and resuspended in PBS, 0.8 N NaOH, and incubated at 30 °C for 36 h. After two washes with distilled H_2_O, the CPS extract was recovered by resuspension in water. The amount of CPS present in the extract was estimated by measuring the sialic acid content using the colorimetric resorcinol-hydrochloric acid method by mixing 120 μL of extract with 380 μL of water and 500 μL of 6% resorcinol solution. After boiling for 20 min, the absorbance was measured at 564 nm.

### Measurements of H_2_O_2_, superoxide dismutase (SOD) and catalase (CAT)

The effect of Aq on SOD and CAT in *A. hydrophila*-infected SGP and TGP cells, or in challenged shrimp, was measured as previously described (Pet et al., 2025). SOD activity was determined using a commercially available SOD colorimetric activity kit (Thermo Fisher, Horsham, United Kingdom), and CAT activity was measured using a catalase activity kit (Abcam, Trumpington, United Kingdom, ab83464). The methods were performed as indicated in the instruction manuals. Inhibitors of NADPH activity, including diphenyleneiodonium chloride (DPI, Sigma; 15 μM, 45 min pre-incubation and wash-out) and bovine liver catalase (Sigma-Aldrich, Gillingham, United Kingdom; 300 U/mL), were used during the 24 h measurement interval. SOD and CAT activity was measured in disrupted gut tissue, sonicated for 60 s (4 × ) at 4 °C (on ice) in 1% saline solution, and then centrifuged at 2,500 rpm at 4 °C for 5 min (Ultrawave DP200-00, Ultrawave Ltd., Cardiff, United Kingdom). Superoxide dismutase (SOD) and catalase (CAT) activity was measured in the resulting supernatants. Hydrogen peroxide (H_2_O_2_) in the WSSV-infected SGP cells was measured using a PeroxiDetect™ Kit (Sigma-Aldrich, Gillingham, United Kingdom). All experiments were performed in triplicate.

### Statistical analysis

Statistical analyses were performed using GraphPad software, version 11. In some cases, data were represented as mean ± SD. *P* < 0.05 were considered statistically significant following estimations using the Student’s *t*-test. One-way ANOVA, Two-way ANOVA, Dunnett’s and Šídák’s tests were used for grouped and multiple comparisons.

## Results

### Determination of inhibitory concentrations and impact on virulence

**FIGURE 1:**
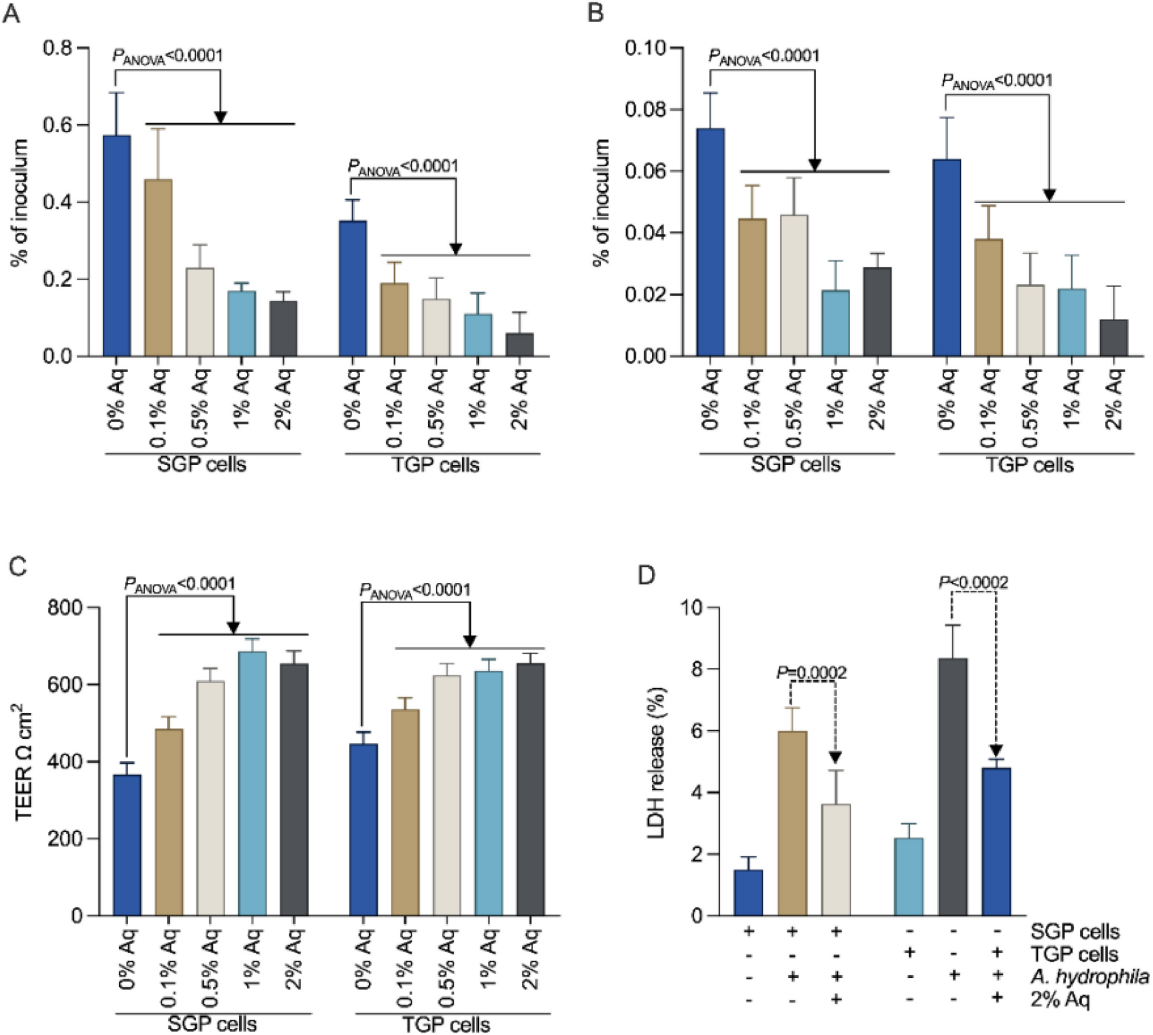
AuraAqua reduces *A. hydrophila* adhesion, invasion, cytotoxicity, and preserves epithelial barrier integrity in SGP and TGP gut primary cells. **(A)** Adhesion of *A. hydrophila* (ATCC 7966) to shrimp gut primary (SGP) and tilapia gut primary (TGP) cells following infection in the presence of increasing concentrations of AuraAqua (0.1, 0.5, 1, and 2%). **(B)** Invasion of SGP and TGP cells by *A. hydrophila* under the same Aq treatment conditions. **(C)** Transepithelial electrical resistance (TEER) of SGP and TGP monolayers measured 3 h post-infection. **(D)** Lactate dehydrogenase (LDH) release from SGP and TGP cells at 3 h post-infection. Data are presented as mean ± SD from at least three independent experiments performed in triplicate. Statistical significance was assessed by one-way ANOVA with Dunnett’s multiple comparisons test; *P*-values are indicated on the graphs.

To assess the effect of Aq on *A. hydrophila* virulence, we first determined the MIC and MBC. Our results indicated an MIC of 1% and an MBC of 3%. Based on these findings, we selected a range of concentrations (0.1, 0.5, 1, and 2% Aq) to further investigate their effect on preventing *A. hydrophila* infection of shrimp gut primary cells (SGP) and Tilapia gut primary cells (TGP). As shown in Figure 1A, these concentrations significantly reduced (*P* < 0.0001) the ability of *A. hydrophila* to attach to both primary SGP and TGP cells. Therefore, Figure 1B shows that the mixture of organic acids also significantly reduced the ability of *A. hydrophila* to penetrate and internalize in both cell lines used (*P* < 0.0001). Furthermore, the impact of AuraAqua on epithelial barrier function (Figure 1C) was evaluated by measuring transepithelial electrical resistance (TEER) in SGP and TGP monolayers post-infection. Treatment with increasing concentrations of Aq (0.1–2%) preserved TEER values relative to control wells infected with *A. hydrophila* alone, indicating that Aq helped maintain cellular integrity and tight junction function. We also demonstrate that, at 3 h post-infection, all Aq concentrations tested significantly (*P* < 0.05) reduced LDH release (Figure 1D) compared to the untreated control, demonstrating its protective effect against *A. hydrophila*-induced cellular damage. These protective effects were consistent across multiple independent experiments and were statistically significant (*P* < 0.05), suggesting that AuraAqua mitigates bacterial disruption of epithelial barriers.

### The impact of AuraAqua on *A. hydrophila* motility and biofilm formation

**FIGURE 2:**
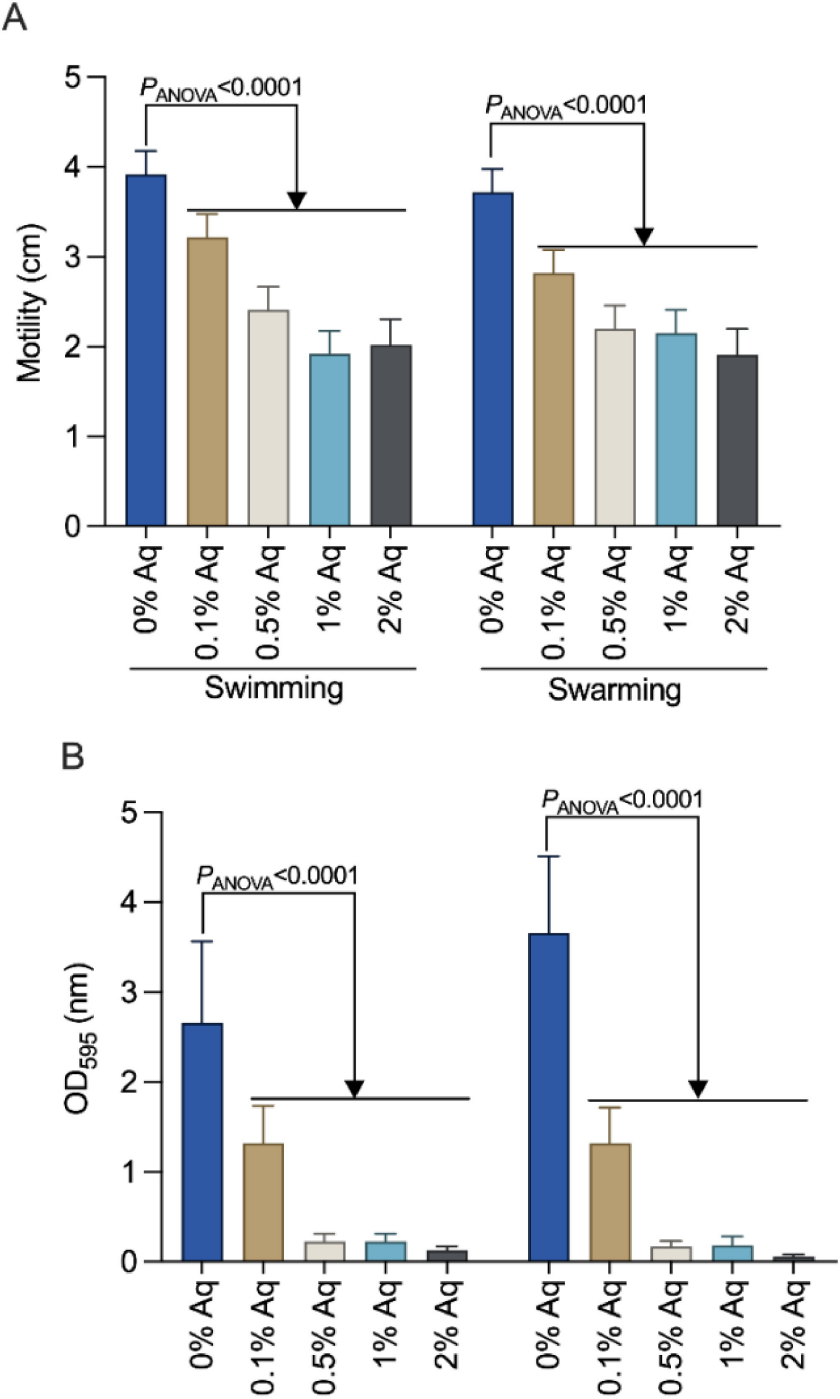
AuraAqua impairs *A. hydrophila* motility and biofilm formation. **(A)** Swimming and swarming motility of *A. hydrophila* after 24 h exposure to increasing concentrations of AuraAqua (0.1, 0.5, 1, and 2%) and **(B)** quantification of biofilm formation. Data are presented as mean ± SD from at least three independent experiments performed in triplicate. Statistical significance was assessed by one-way ANOVA; *P*-values are indicated on the graphs.

To further investigate the observed inhibitory effect on *A. hydrophila* adhesion and internalization in SGP and TGP cells, we next tested the impact of 0.1, 0.5, 1, and 2% Aq on bacterial motility and biofilm formation. Swimming and swarming assays (Figure 2A) showed that increasing concentrations of Aq significantly reduced the motility of *A. hydrophila* at all tested concentrations (*P* < 0.0001). Furthermore, quantitative biofilm assays revealed dose-dependent inhibition of biofilm formation, with notable decreases (*P* < 0.0001) at concentrations as low as 0.5% (Figure 2B). Results showed a dose-dependent reduction in both motility and biofilm density with increasing Aq concentrations, indicating a significant impairment of the bacterium’s ability to colonize surfaces and establish persistent infections.

### AuraAqua directly controls *hcp1* expression and the virulence of *A. hydrophila* without affecting *TonB* expression

**FIGURE 3:**
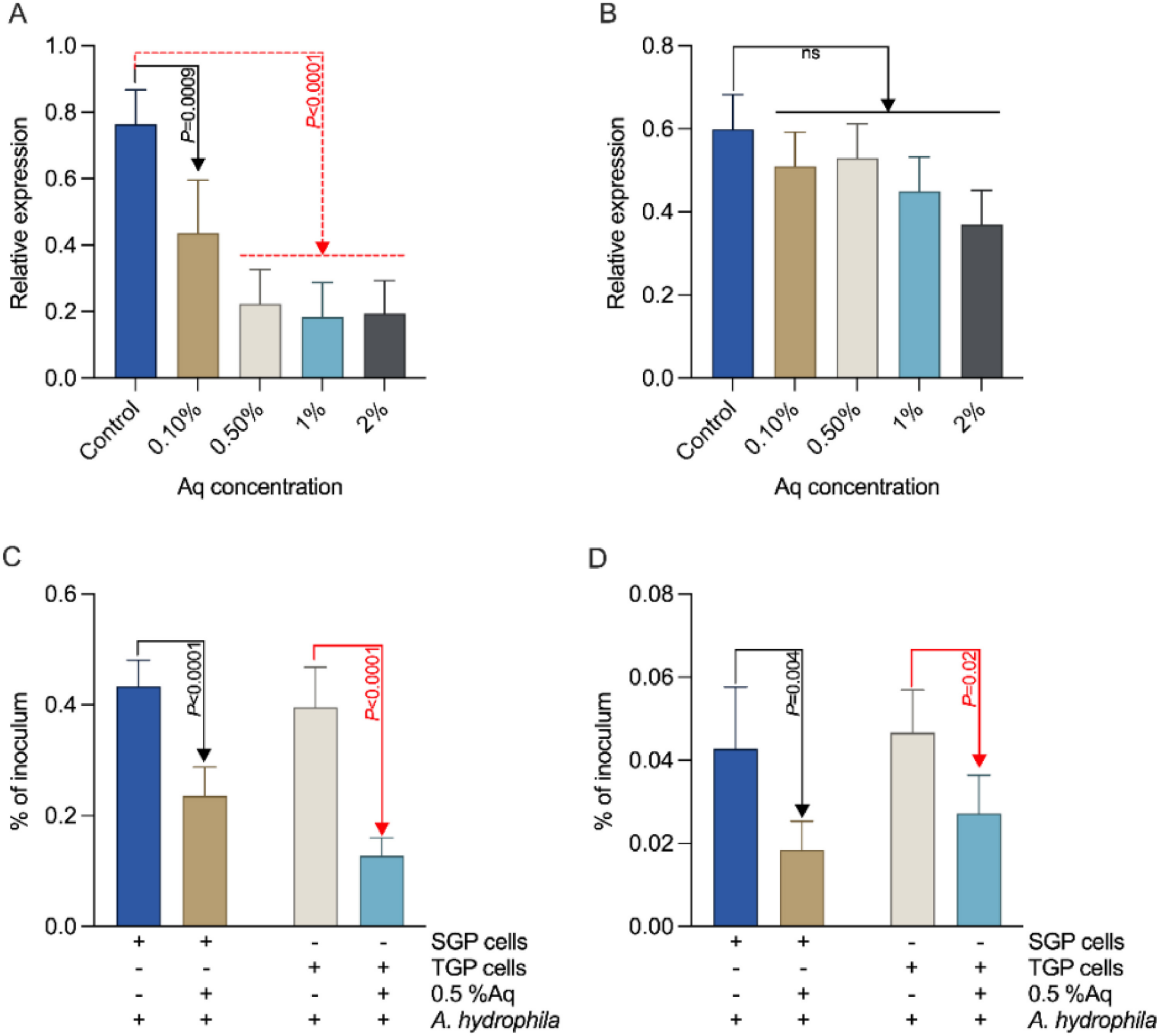
AuraAqua downregulates *hcp1* and reduces *A. hydrophila* adhesion and invasion without affecting *TonB* expression. **(A)** Relative mRNA expression of the virulence gene *hcp1* in *A. hydrophila* grown for 24 h in the presence of increasing concentrations of AuraAqua (0.1, 0.5, 1, and 2%) compared to the untreated control. **(B)** Relative mRNA expression of *TonB* under the same conditions. Adhesion **(C)** and invasion **(D)** of SGP and TGP cells by *A. hydrophila* grown for 24 h in in the presence of 0.5% AuraAqua, compared with untreated bacteria. Relative gene expression was calculated using the 2^–ΔΔCt^ method. Data are presented as mean ± SD from at least three independent experiments performed in triplicate. Statistical significance was determined using one-way ANOVA with Šídák’s multiple comparisons test; *P-*values are indicated on the graphs.

To determine whether the observed effects on virulence were associated with changes in gene expression, the transcriptional levels of *hcp1* and *TonB* were quantified by qRT-PCR after Aq treatment. We show that Aq significantly downregulated *hcp1* (Figure 3A) expression (*P* < 0.05) but not *TonB* (Figure 3B) at concentrations between 0.1 and 2%. Furthermore, we show that *A. hydrophila* grown in the presence of 0.5% Aq for 24 h was significantly (*P* < 0.05) less able to adhere to both SGP (Figure 3C) and TGP cells (Figure 3D). These results demonstrated that downregulation of *hcp1* expression in *A. hydrophila* exposed to Aq, whereas *TonB* expression remained unchanged across all concentrations tested. This suggests that Aq specifically targets the *hcp1*-mediated virulence pathway without broadly affecting *TonB*-mediated iron acquisition.

### AuraAqua exerts a targeted rather than broad gene expression modulation

**FIGURE 4:**
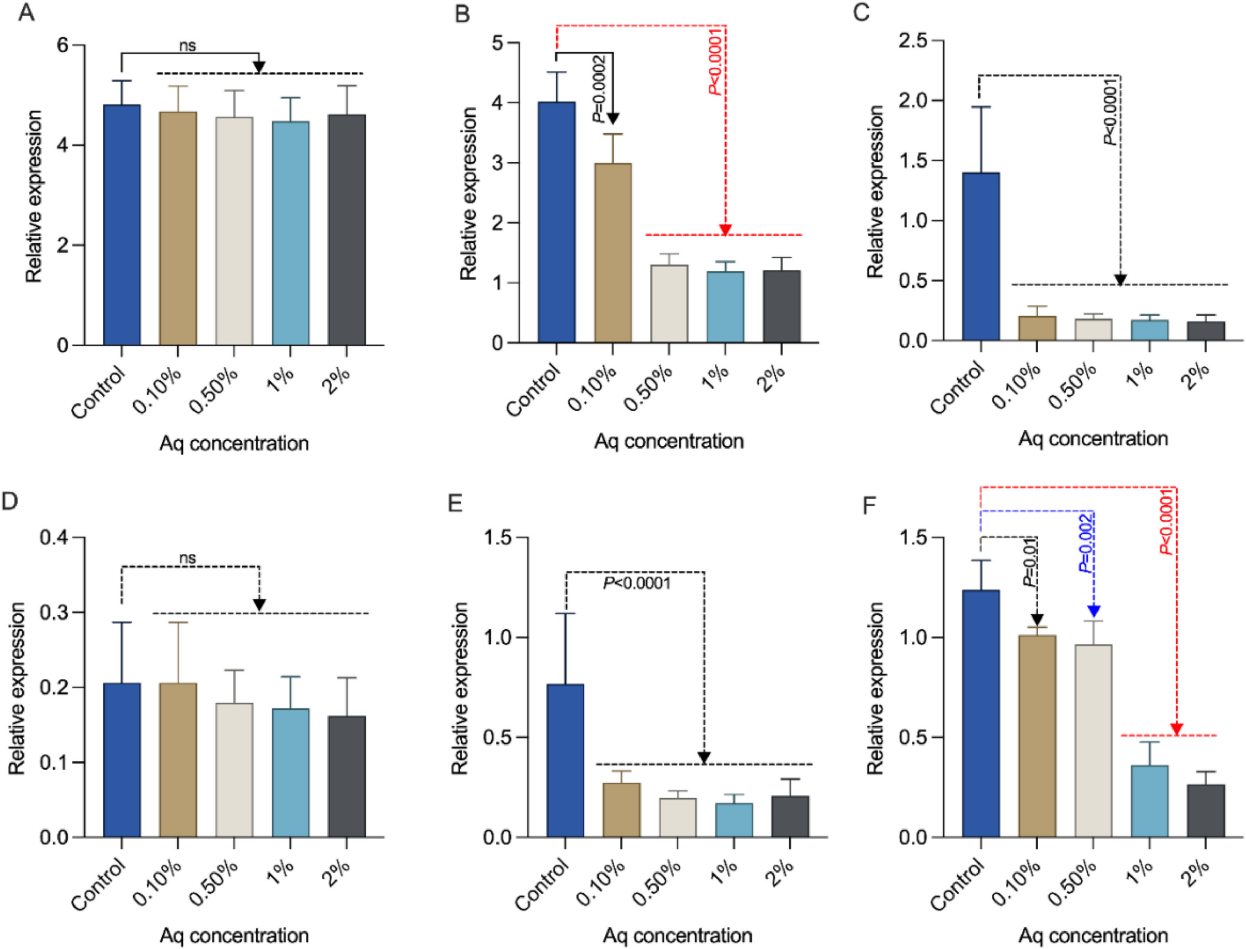
AuraAqua selectively modulates *A. hydrophila* virulence gene expression. Relative mRNA expression levels of **(A)** *aerA*, **(B)** *act*, **(C)** *fla*, **(D)** *alt*, **(E)** *hlyA*, and **(F)** *acg* in *A. hydrophila* following 24 h growth in the presence of increasing concentrations of AuraAqua (0.1, 0.5, 1, and 2%) compared with untreated controls. Relative expression was calculated using the 2^–ΔΔCt^ method. Data are presented as mean ± SD from at least three independent experiments performed in triplicate. Statistical significance was determined using one-way ANOVA with Šídák’s test for multiple comparisons. *P*-values are indicated on the graphs.

Our next aim was to investigate whether the lack of effect on *TonB* expression reflects targeted modulation of bacterial gene expression rather than broad suppression of gene activity. To achieve this, we have analyzed the expression of *aerA* (Figure 4A)*, act* (Figure 4B)*, fla* (Figure 4C)*, alt* (Figure 4D)*, hlyA* (Figure 4E), and *acg* (Figure 4F) genes following growth of *A. hydrophila* in the presence of 0.1–2% Aq for 24 h. Quantitative analysis revealed that exposure to Aq resulted in marked changes in the transcriptional activity of virulence-associated genes, specifically *act*, *fla*, *hlyA*, and *acg*, across all concentrations investigated. As observed for *TonB* expression, the presence of the organic acid mixture had no effect on the expression of *aerA* and *alt* across the concentrations investigated. These findings suggest that Aq may exert its anti-virulence effects by interfering with specific molecular pathways, thereby limiting the bacterium’s ability to adhere, invade, and persist within host cells.

### Growth of *A. hydrophila* in the presence of AuraAqua leads to CPS loss

**FIGURE 5:**
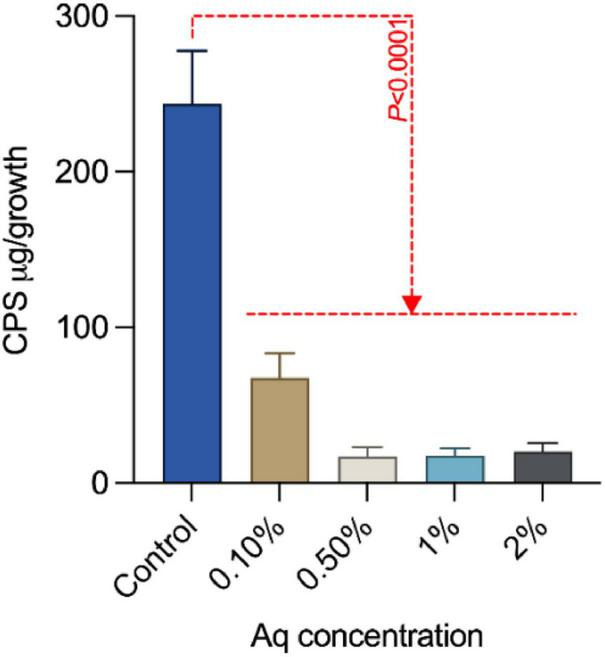
Effect of AuraAqua on capsular polysaccharide (CPS) production in *A. hydrophila*. CPS levels were measured spectrophotometrically after 24 h of exposure to AuraAqua at increasing concentrations (0.1, 0.5, 1, and 2%). Results show a significant dose-dependent reduction in CPS, indicating compromised bacterial structural integrity and reduced potential for immune evasion. Error bars represent standard deviations; statistical significance was assessed by one-way ANOVA, with *P* values indicated on the graph. Experiments were performed in triplicate.

To further investigate the effect of Aq on the structural integrity of *A. hydrophila*, we examined the production of capsular polysaccharide (CPS) after 24 h of exposure to varying concentrations of Aq (0.1–2%). Our results showed a significant reduction in CPS levels (*P* < 0.0001), as measured spectrophotometrically (Figure 5). This loss of CPS is consistent with a diminished capacity for immune evasion and may further contribute to the decreased virulence observed in treated bacteria.

### The loss of *A. hydrophila* CPS is induced by AuraAqua during infection

**FIGURE 6:**
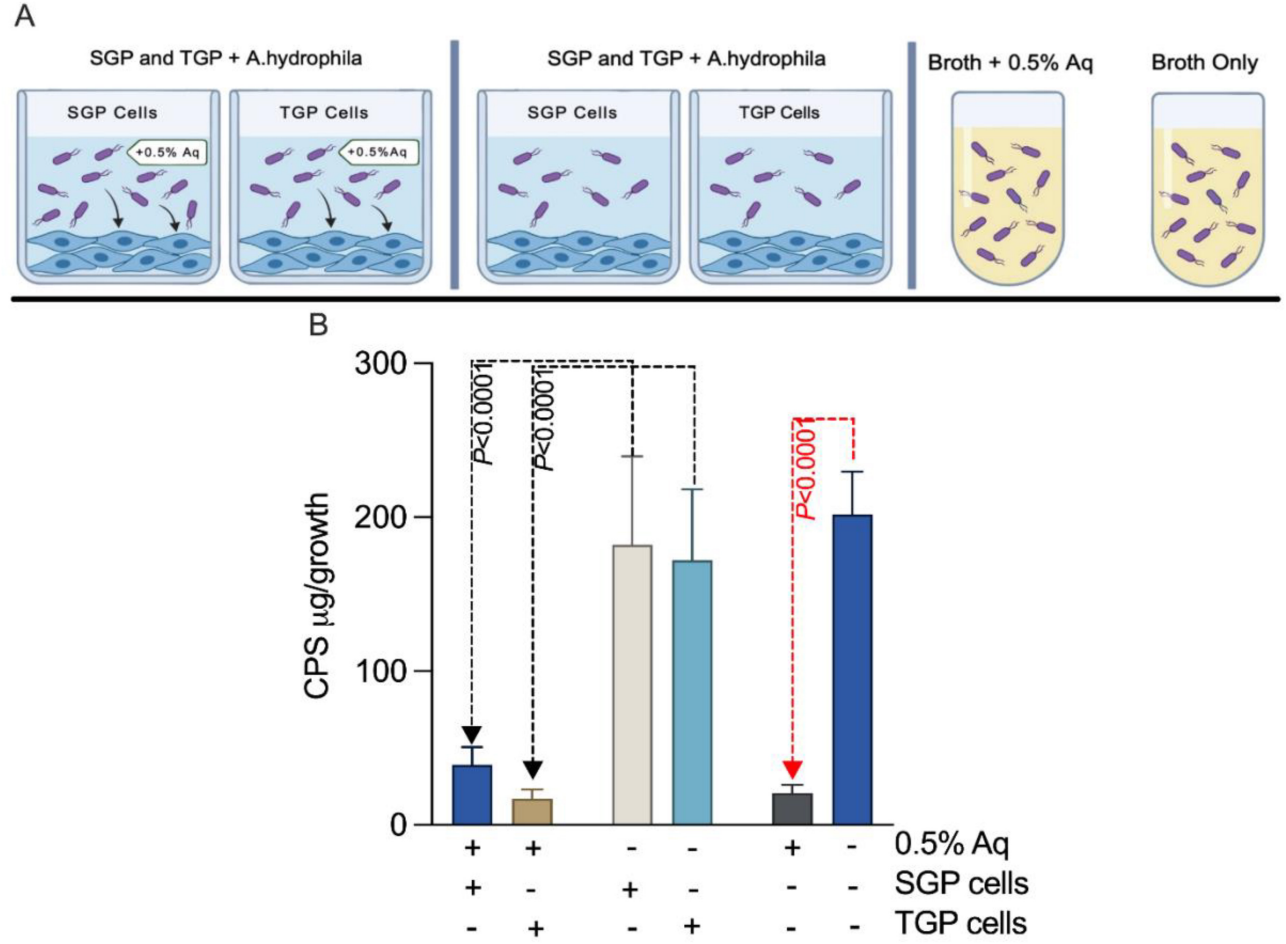
AuraAqua induces CPS loss in *A. hydrophila* during infection of SGP and TGP cells. **(A)** schematic overview of the experimental design used to assess capsular polysaccharide (CPS) production by *A. hydrophila* during infection 0.5% AuraAqua. **(B)** quantification of CPS produced by *A. hydrophila* during infection of SGP cells with or without AuraAqua treatment. Data are presented as mean ± SD from at least three independent experiments performed in triplicate. Statistical significance was determined using one-way ANOVA with Šídák’s test for multiple comparisons. *P*-values are indicated on the graphs.

Next, we have designed an experiment to investigate whether Aq-induced capsule loss occurs prior to infection in the presence of SGP and TGP cells (Figure 6A). To achieve this, CPS was measured in bacteria isolated from infection supernatants, with or without a subinhibitory concentration of 0.5% Aq, and in bacteria grown in bacterial broth, with or without 0.5% Aq. The 0.5% concentration was chosen as the key working concentration because it is the highest non-growth-inhibitory dose that still produced robust and reproducible anti-virulence effects and had a significant impact on CPS production as described in Figure 5. Capsular polysaccharide (CPS) production was quantified spectrophotometrically after 3 h. The experiments were performed in the presence of 0.5% Aq, and after 3 h, the bacteria from the infection supernatants were used to quantify CPS. After incubation, we observed a significant decrease (*P* < 0.0001) in CPS production in *A. hydrophila* exposed to 0.5% Aq during infection compared with untreated controls (Figure 6B). This was evident in both SGP and TGP cell infection models, indicating that Aq-induced capsule loss is not limited to *in vitro* bacterial cultures but persists during host cell interactions. These findings reinforce Aq’s potential as an effective agent for attenuating bacterial virulence by disrupting capsule formation, thereby weakening the pathogen’s ability to evade immune responses and establish infection.

### AuraAqua reduces oxidative stress in infected SGP and TGP cells

**FIGURE 7:**
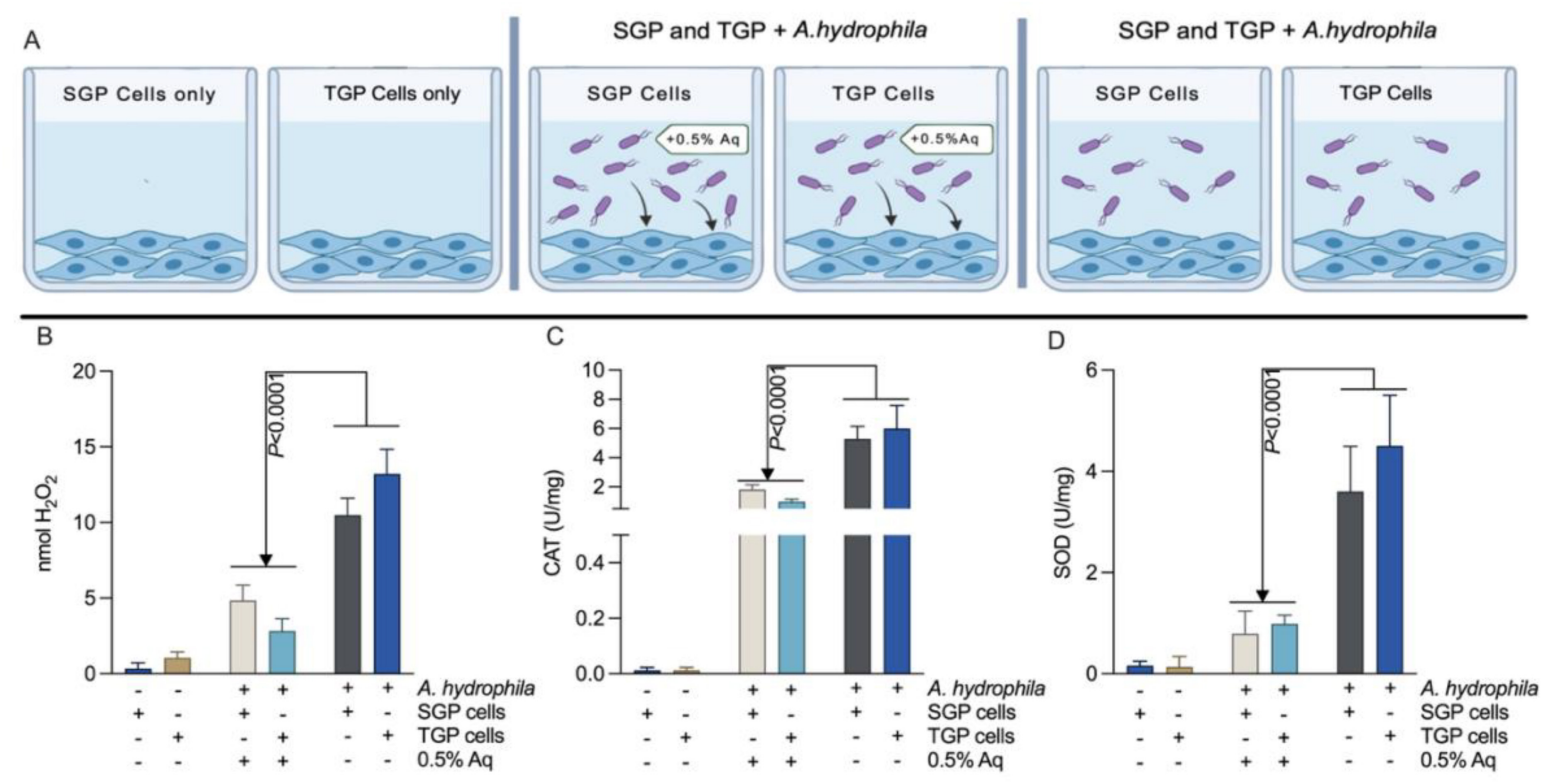
AuraAqua reduces oxidative stress responses in *A. hydrophila*-infected SGP and TGP cells. Uninfected, untreated cells served as controls. **(A)** schematic overview of the experimental design. **(B)** H2O2, **(C)** CAT, and **(D)** SOD production levels in the presence of 0.5% AuraAqua. Data are presented as mean ± SD from at least three independent experiments performed in triplicate. Statistical significance was determined using one-way ANOVA with ídák’s test for multiple comparisons. *P* values are indicated on the graphs.

The decrease in infection prompted us to investigate whether oxidative stress also diminished during infection (Figure 7). Hydrogen peroxide (H_2_O_2_) levels, superoxide dismutase (SOD) activity, and catalase (CAT) activity were measured in primary shrimp gut (SGP) and primary tilapia gut (TGP) cells after infection with *A. hydrophila*, in the absence or presence of 0.5% Aq. Treatment with Aq significantly (*P* < 0.0001) lowered the production of these oxidative stress markers in both cell types compared with untreated infected controls. This indicates that Aq not only reduces bacterial virulence but also alleviates host cell oxidative stress, potentially enhancing cellular resilience during infection.

## Discussion

Blends of organic acids alone (Butucel et al., 2022) or in combination with essential oils (He et al., 2017) have been shown to positively affect the intestinal microflora of shrimp or fish and to elicit an immune response during bacterial infection. Almost 30 years ago, it was determined that organic acids (lactic, citric, acetic, tartaric) can be effective in controlling the growth kinetics of *A. hydrophila* in culture at various temperatures (5–19°C) (Palumbo et al., 1992). Recent studies have shown that lactic acid can enhance the activity of fish endogenous antimicrobial peptides (Epinedicin-1 and Pediocin PA-1) and cause bacterial membrane damage and increased release of nucleic acids and proteins in *A. hydrophila* (Wang et al., 2020; Li et al., 2024). Citric acid, another component of Aq, was also shown to enhance the activity of Azomite (hydrated aluminosilicate) and effectively improve the intestinal morphology and microbial flora in juvenile largemouth bass infected with *A. hydrophila* (Zhang et al., 2023). Organic acids (phenyllactic acid—PLA) produced by probiotic bacteria (*Leuconostoc mesenteroides*) can have similar effects by reducing *A. hydrophila* surface polysaccharide (CPS) production and biofilm formation, resulting in lower intestinal colonization levels in the fish gut and improved intestinal integrity (Xia et al., 2025). In our SGP and TGP infection models, Aq reduced CPS production and the virulence of *A. hydrophila*, and significantly diminished the bacterium’s motility and biofilm-forming abilities. We demonstrate that their effect directly triggers the bacterium, even in the presence of SGP and TGP cells.

Existing data show that lactic and malic acid treatments impair *A. hydrophila’s* ability to form biofilms by affecting motility, bacterial protein synthesis, membrane function, and flagella through their effects on the expression of *hcp*1 and *TonB* (Dong et al., 2023; Wang et al., 2023). In our study, the organic acid mixture (Aq) suppresses the expression of the *hcp1* gene but not significantly that of *TonB* in *A. hydrophila*. Hcp, one of the effector proteins of the Type 6 Secretion System (T6SS) in *A. hydrophila*, was shown to clearly influence bacterial motility, protease production and biofilm formation, and to have a direct virulent function during infection in both *in vitro* and *in vivo* models (Sha et al., 2013). The application of a lactic acid-malic acid complex solution notably decreased *A. hydrophila* counts by 4.16 log CFU/mL and caused significant damage to the bacterial membrane, as observed through scanning electron microscopy (SEM) (Huang et al., 2025).

The hemolysin (*hlyA*) and aerolysin (*aerA*) genes in *A. hydrophila* are both associated with synergistic activity that affects bacterial virulence (Yu et al., 2005). Interestingly, in our study, the organic acid mixture (Aq) downregulated *hlyA*, but not *aerA*, with a significant effect on the bacterium’s ability to infect SGP and TGP cells. We have previously shown that Aq downregulated hemolysin-related genes in *Lactococcus garvieae* and attenuated haemolysis in fish red blood cells (Balta et al., 2025). One reason we observed lower bacterial motility and, consequently, attenuated virulence in our study is the decrease in *fla* gene mRNA levels in the presence of Aq. In *A. hydrophila*, motility and flagella play a key role in virulence, and flagellin (*fla*) plays a key role (Rabaan et al., 2001). The *act* gene (cytotoxic enterotoxin gene) is crucial during *A. hydrophila* infection (Sha et al., 2001) by mediating inflammation and apoptosis (Galindo et al., 2003). In our study, the most dramatic downregulation in *act* expression was observed at concentrations ranging from 0.5 to 2% Aq.

*A. hydrophila* infection induces oxidative stress, reflected by elevated superoxide dismutase (SOD) activity in tissues such as the intestine, liver, and gills during early infection stages in fish (Chen et al., 2020) and crabs (Zheng et al., 2022). In our study, using primary gut cells from shrimp and fish, we similarly observed increased SOD and catalase (CAT) activities in infected SGP and TGP cells. However, treatment with Aq significantly reduced these oxidative stress markers, likely due to its suppression of bacterial virulence factors and consequent reduction in infection severity. The CAT and SOD activity profiles may reflect cell-type specificity, consistent with the well-documented species-dependent variation in the expression of these enzymes (Bandeira Junior and Baldisserotto, 2021). The reduction in oxidative stress was further evidenced by the markedly lower H_2_O_2_ levels observed in the presence of Aq. Furthermore, our results highlight the broad spectrum of Aq’s efficacy, which not only targets the structural components of *A. hydrophila* but also modulates the host response to infection. This dual action, attenuating bacterial defenses while reducing host oxidative stress, suggests that organic acid mixtures, such as Aq, could serve as promising adjuncts to current preventive strategies in aquaculture. Future research should focus on optimizing dosing regimens and exploring synergistic effects with existing treatments to enhance disease resistance and promote overall health in aquatic species.

## Conclusion

This study demonstrates that the organic acid mixture AuraAqua (Aq) exerts a potent anti-virulence effect against *A. hydrophila* in primary gut cells from shrimp and tilapia. At sub-inhibitory concentrations, Aq significantly reduced bacterial adhesion and invasion, preserved epithelial barrier integrity, and lowered cytotoxicity, as indicated by sustained TEER values and reduced LDH release.

**FIGURE 8:**
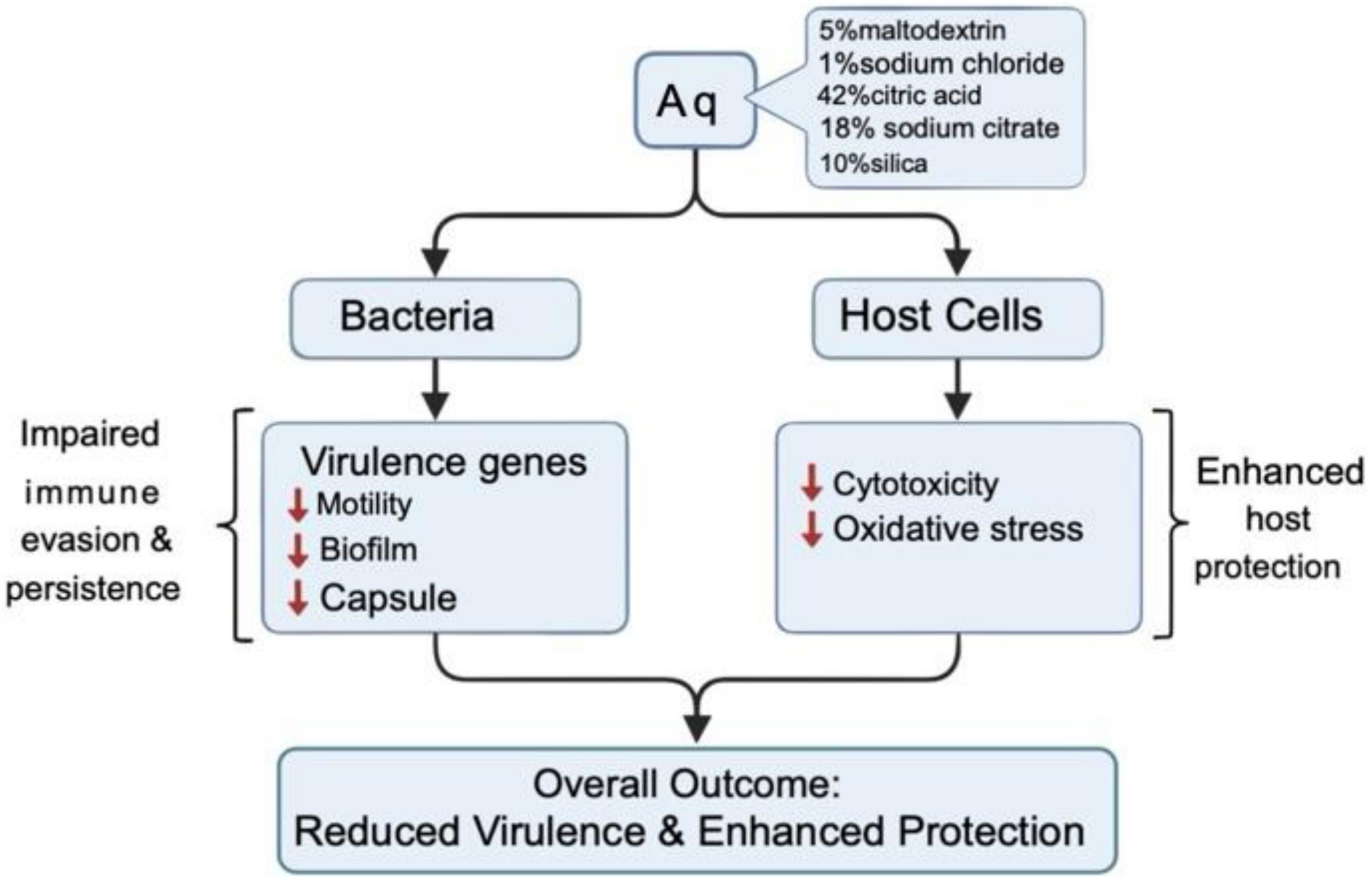
Schematic mechanism of action.

The mechanism of action involves significant impairment of motility and biofilm formation, indicating a decreased ability of *A. hydrophila* to colonize host tissues and establish persistent infections (Figure 8). At the molecular level, Aq selectively altered the expression of key virulence-associated genes, including *hcp1, act, fla, hlyA*, and *acg*, while largely leaving *TonB*, *aerA*, and alt unaffected. Simultaneously, Aq markedly decreased CPS production both *in vitro* and during infection, further impairing immune evasion mechanisms and likely reducing bacterial fitness within the host environment. Importantly, Aq also decreased H2O2 levels and SOD and CAT activities. This dual effect, attenuation of bacterial virulence alongside modulation of host oxidative responses, positions Aq as a promising non-antibiotic intervention for enhancing gut health and disease resistance in aquaculture systems. Future *in vivo* studies across different species, production conditions, and dosing regimens are needed to confirm these benefits at the farm level and to investigate potential synergistic effects with other natural antimicrobials and current health management strategies.

## Data Availability

The raw data supporting the conclusions of this article will be made available by the authors, without undue reservation.

## References

[B1] Abdul Kari Z. Wee W. Mohamad Sukri S. A. Che Harun H. Hanif Reduan M. F. Irwan Khoo M. et al. (2022). Role of phytobiotics in relieving the impacts of Aeromonas hydrophila infection on aquatic animals: A mini-review. *Front. Vet. Sci.* 9:1023784. 10.3389/fvets.2022.1023784 36277060 PMC9582345

[B2] Balta I. Simiz F. D. Stef D. Pet I. Dumitrescu G. Iancu T. et al. (2025). A new strategy to prevent emerging Lactococcus garvieae infections by using organic acids as antimicrobials in vitro and ex vivo. *Int. J. Mol. Sci.* 26:3423. 10.3390/ijms26073423 40244309 PMC11989770

[B3] Balta I. Stef L. Butucel E. Gradisteanu Pircalabioru G. Venig A. Ward P. et al. (2022). The antioxidant effect of natural antimicrobials in shrimp primary intestinal cells infected with Nematopsis messor. *Antioxidants* 11:974. 10.3390/antiox11050974 35624838 PMC9137680

[B4] Bandeira Junior G. Baldisserotto B. (2021). Fish infections associated with the genus Aeromonas: A review of the effects on oxidative status. *J. Appl. Microbiol.* 131 1083–1101. 10.1111/jam.14986 33382188

[B5] Butucel E. Balta I. McCleery D. Marcu A. Stef D. Pet I. et al. (2022). The prebiotic effect of an organic acid mixture on Faecalibacterium prausnitzii metabolism and its anti-pathogenic role against vibrio parahaemolyticus in Shrimp. *Biology* 12:57. 10.3390/biology12010057 36671749 PMC9855566

[B6] Chen J. Liu N. Zhang H. Zhao Y. Cao X. (2020). The effects of Aeromonas hydrophila infection on oxidative stress, nonspecific immunity, autophagy, and apoptosis in the common carp. *Dev. Comp. Immunol.* 105:103587. 10.1016/j.dci.2019.103587 31875516

[B7] Chen Q. Zhang Z. Tang H. Zhou L. Ao S. Zhou Y. et al. (2021). Aeromonas hydrophila associated with red spot disease in Macrobrachium nipponense and host immune-related gene expression profiles. *J. Invertebr. Pathol.* 182:107584. 10.1016/j.jip.2021.107584 33811849

[B8] da Silva E. G. Bandeira Junior G. Cargnelutti J. F. Santos R. C. V. Gündel A. Baldisserotto B. (2021). In vitro antimicrobial and antibiofilm activity of S-(-)-limonene and R-(+)-limonene against fish bacteria. *Fishes* 6:32. 10.3390/fishes6030032

[B9] Dong J. Li S. Zhou S. Liu Y. Yang Q. Xu N. et al. (2025a). Novel insights into the therapeutic effect of amentoflavone against aeromonas hydrophila infection by blocking the activity of aerolysin. *Int. J. Mol. Sci.* 26:2370. 10.3390/ijms26052370 40076989 PMC11900166

[B10] Dong J. Tong J. Li S. Ma X. Yang Q. Liu Y. et al. (2025b). Turmeric oil interferes with quorum sensing as an alternative approach to control aeromonas hydrophila infection in aquaculture. *Biology* 14:483. 10.3390/biology14050483 40427672 PMC12108673

[B11] Dong Y. Xu M. Wan X. Zhao D. Geng J. Huang H. et al. (2023). TonB systems are required for Aeromonas hydrophila motility by controlling the secretion of flagellin. *Microbes Infect.* 25:105038. 10.1016/j.micinf.2022.105038 35963567

[B12] Galindo C. L. Sha J. Ribardo D. A. Fadl A. A. Pillai L. Chopra A. K. (2003). Identification of Aeromonas hydrophila cytotoxic enterotoxin-induced genes in macrophages using microarrays. *J. Biol. Chem.* 278 40198–40212. 10.1074/jbc.M305788200 12824169

[B13] García-Pérez J. Pérez-Sabino J. Mendoza-Elvira S. Ribeiro, da Silva A. J. Ulloa-Rojas J. (2024). Antimicrobial activity of diverse chemotypes of Lippia graveolens against aeromonas hydrophila isolated from Oreochromis niloticus. *Uniciencia* 38 551–566. 10.15359/ru.38-1.30

[B14] He W. Rahimnejad S. Wang L. Song K. Lu K. Zhang C. (2017). Effects of organic acids and essential oils blend on growth, gut microbiota, immune response and disease resistance of Pacific white shrimp (Litopenaeus vannamei) against Vibrio parahaemolyticus. *Fish. Shellfish Immunol.* 70 164–173. 10.1016/j.fsi.2017.09.007 28882791

[B15] Huang H. Tong Y. Lyu X. Zhao W. Yang R. (2025). Ultrasound and lactic/malic acid treatment for mitten crab decontamination: Efficacy and mechanisms against A. hydrophila. *Ultrason Sonochem.* 115:107294. 10.1016/j.ultsonch.2025.107294 40023899 PMC11919395

[B16] Lamy B. Baron S. Barraud O. (2022). Aeromonas: The multifaceted middleman in the One Health world. *Curr. Opin. Microbiol.* 65 24–32. 10.1016/j.mib.2021.09.012 34717260

[B17] Li S. Zhou S. Yang Q. Liu Y. Yang Y. Xu N. et al. (2023). Cinnamaldehyde decreases the pathogenesis of aeromonas hydrophila by inhibiting quorum sensing and biofilm formation. *Fishes* 8:122. 10.3390/fishes8030122

[B18] Li Y. Wang Y. Luo Y. L. Bai D. Q. Zhang G. Wang J. R. et al. (2024). Epinecidin-1 and lactic acid synergistically inhibit Aeromonas hydrophila through membrane disruption. *Microb. Pathog.* 196:106879. 10.1016/j.micpath.2024.106879 39218372

[B19] Liliana P. C. Dumitrescu G. McCleery D. Pet I. Iancu T. Stef L. et al. (2024). Organic acids mitigate Streptococcus agalactiae virulence in Tilapia fish gut primary cells and in a gut infection model. *Ir. Vet. J.* 77:10. 10.1186/s13620-024-00272-1 38797844 PMC11129440

[B20] Méndez L. R. Rodríguez-Cornejo T. Rodríguez-Ramos T. Al-Hussinee L. Velázquez J. Campbell J. H. et al. (2024). PACAP sequence modifications modulate the peptide antimicrobial activity against bacterial pathogens affecting aquaculture. *Fish Shellf. Immunol.* 148:109512. 10.1016/j.fsi.2024.109512 38499216

[B21] Palumbo S. A. Williams A. C. Buchanan R. L. Phillips J. G. (1992). Model for the anaerobic growth of aeromonas hydrophila K144. *J. Food Prot.* 55 260–265. 10.4315/0362-028X-55.4.260 31071791

[B22] Peng Z. Wei C. Li B. Chen M. Wang H. Zou Z. et al. (2025). ClavF derived from Clavanins as a promising candidate for fighting infections from multiple-drug resistance Aeromonas hydrophila. *Fish Shellf. Immunol.* 165:110488. 10.1016/j.fsi.2025.110488 40505880

[B23] Pet I. Balta I. Corcionivoschi N. Iancu T. Stef D. Stef L. et al. (2025). Shrimp white spot viral infections are attenuated by organic acids by regulating the expression of HO-1 oxygenase and beta-1,3-glucan-binding protein. *Antioxidants* 14:89. 10.3390/antiox14010089 39857423 PMC11763281

[B24] Rabaan A. A. Gryllos I. Tomás J. M. Shaw J. G. (2001). Motility and the polar flagellum are required for Aeromonas caviae adherence to HEp-2 cells. *Infect. Immun.* 69 4257–4267. 10.1128/iai.69.7.4257-4267.2001 11401962 PMC98495

[B25] Saad M. F. Elsayed M. M. Khder M. Abdelaziz A. S. El-Demerdash A. S. (2024). Biocontrol of multidrug resistant pathogens isolated from fish farms using silver nanoparticles combined with hydrogen peroxide insight to its modulatory effect. *Sci. Rep.* 14:7971. 10.1038/s41598-024-58349-4 38575637 PMC10994946

[B26] Semwal A. Kumar A. Kumar N. (2023). A review on pathogenicity of Aeromonas hydrophila and their mitigation through medicinal herbs in aquaculture. *Heliyon* 9:e14088. 10.1016/j.heliyon.2023.e14088 36938468 PMC10018484

[B27] Sha J. Lu M. Chopra A. K. (2001). Regulation of the cytotoxic enterotoxin gene in Aeromonas hydrophila: Characterization of an iron uptake regulator. *Infect. Immun.* 69 6370–6381. 10.1128/IAI.69.10.6370-6381.2001 11553581 PMC98772

[B28] Sha J. Rosenzweig J. A. Kozlova E. V. Wang S. Erova T. E. Kirtley M. L. et al. (2013). Evaluation of the roles played by Hcp and VgrG type 6 secretion system effectors in Aeromonas hydrophila SSU pathogenesis. *Microbiology* 159 1120–1135. 10.1099/mic.0.063495-0 23519162 PMC3709694

[B29] Tao L.-T. Gao D.-P. Liu Y.-L. Sun W.-W. Shan X.-F. (2026). The genus aeromonas in aquaculture: A comprehensive review of prevalence, virulence, and antibiotic resistance with an emphasis on key pathogenic species. *Rev. Aquacult.* 18:e70097. 10.1111/raq.70097

[B30] Urgesa G. Lu L. Gao J. Guo L. Qin T. Liu B. et al. (2024). Natural sunlight-mediated emodin photoinactivation of aeromonas hydrophila. *Int. J. Mol. Sci.* 25:5444. 10.3390/ijms25105444 38791482 PMC11121522

[B31] Wang Y. D. Gong J. S. Guan Y. C. Zhao Z. L. Cai Y. N. Shan X. F. (2023). OmpR (TCS response regulator) of Aeromonas veronii plays a major role in drug resistance, stress resistance and virulence by regulating biofilm formation. *Microb. Pathog.* 181:106176. 10.1016/j.micpath.2023.106176 37244492

[B32] Wang Y. Wang J. Bai D. Wei Y. Sun J. Luo Y. et al. (2020). Synergistic inhibition mechanism of pediocin PA-1 and L-lactic acid against Aeromonas hydrophila. *Biochim. Biophys. Acta Biomembr.* 1862:183346. 10.1016/j.bbamem.2020.183346 32428447

[B33] Xia X. Lin Y. Wang Y. Qin L. Liu S. Zhang H. et al. (2025). Antibacterial mechanisms of PLA on Aeromonas hydrophila and its application in fish disease resistance and fish preservation. *Food Biosci.* 65:106064. 10.1016/j.fbio.2025.106064

[B34] Xu L. Kang X. Wang Z. Xiao Z. Luo Y. (2025). Genomic insights into the pathogenicity of hypervirulent aeromonas hydrophila strain D4 isolated from diseased blunt snout bream with the epidemic sequence type 251 clones. *Pathogens* 14:570. 10.3390/pathogens14060570 40559578 PMC12195866

[B35] Xu T. Rasmussen-Ivey C. R. Moen F. S. Fernández-Bravo A. Lamy B. Beaz-Hidalgo R. et al. (2023). A global survey of hypervirulent aeromonas hydrophila (vAh) identified vAh strains in the lower mekong river basin and diverse opportunistic pathogens from farmed fish and other environmental sources. *Microbiol. Spectr.* 11:e0370522. 10.1128/spectrum.03705-22 36815836 PMC10101000

[B36] Yu H. B. Zhang Y. L. Lau Y. L. Yao F. Vilches S. Merino S. et al. (2005). Identification and characterization of putative virulence genes and gene clusters in Aeromonas hydrophila PPD134/91. *Appl. Environ. Microbiol.* 71 4469–4477. 10.1128/aem.71.8.4469-4477.2005 16085838 PMC1183340

[B37] Zhang C. Chen F. Bai Y. Dong X. Meng X. Wang K.-J. (2024). A novel antimicrobial peptide Spasin141-165 identified from Scylla paramamosain exhibiting protection against Aeromonas hydrophila infection. *Aquaculture* 591:741137. 10.1016/j.aquaculture.2024.741137

[B38] Zhang Y. Huang H. Chang W. T. H. Li X. Leng X. (2023). The combined supplementation of AZOMITE and citric acid promoted the growth, intestinal health, antioxidant, and resistance against Aeromonas hydrophila for largemouth bass, Micropterus salmoides. *Aquac. Nutr.* 2023:5022456. 10.1155/2023/5022456 37881475 PMC10597733

[B39] Zheng N. Wang N. Wang Z. Y. Abdallah G. Zhang B. Y. Wang S. et al. (2022). Effect of infection with Aeromonas hydrophila on antioxidant capacity, inflammation response, and apoptosis proteins in Chinese mitten crab (Eriocheir sinensis). *Comp. Biochem. Physiol. C Toxicol. Pharmacol.* 252:109220. 10.1016/j.cbpc.2021.109220 34718187

[B40] Zhong W. Chen K. Yang L. Tang T. Jiang S. Guo J. et al. (2022). Essential oils from citrus unshiu marc. Effectively kill aeromonas hydrophila by destroying cell membrane integrity, influencing cell potential, and leaking intracellular substances. *Front. Microbiol.* 13:869953. 10.3389/fmicb.2022.869953 35836415 PMC9274202

[B41] Zhong W. Tang P. Liu T. Zhao T. Guo J. Gao Z. (2021). Linalool nanoemulsion preparation, characterization and antimicrobial activity against aeromonas hydrophila. *Int. J. Mole. Sci.* 22:11003. 10.3390/ijms222011003 34681662 PMC8538616

